# Nanocomposites for Environmental and Energy Applications

**DOI:** 10.3390/nano11020345

**Published:** 2021-01-31

**Authors:** Pei Sean Goh, Ahmad Fauzi Ismail

**Affiliations:** Advanced Membrane Technology Research Centre, School of Chemical and Energy Engineering, Universiti Teknologi Malaysia, Johor Bahru 81310, Malaysia; peisean@petroleum.utm.my

Global environmental and energy issues are the two major challenges of the 21st century. Climate change and the energy crisis have urgently called for the establishment of sustainable and innovative solutions to tackle these issues. Among the many established strategies, the emergence of functional nanocomposites as potential tools to address environmental and energy-related issues has attracted wide attention in both laboratory research and industry applications. The synergistic effects concertedly contributed by a broad range of nanomaterials and their host materials have rendered unprecedented advantages over the conventionally used materials. With the astonishing chemical and physical properties exhibited by various nanocomposites, their potentials have been unlocked in many practical applications. Nanocomposites are also particularly interesting in offering sustainable solutions to curb global challenges related to environmental issues and energy shortages.

This Special Issue, themed “Nanocomposites for Environmental and Energy Applications”, gathers material scientists and engineers working in this realm to look into various aspects associated to the development of the state-of-the-art nanocomposites dedicated for environmental and energy applications. A collection of research articles and reviews, with special attentions focused on the synthesis, design, optimization and applications of novel nanocomposites has been compiled in this Special Issue. The nine contributions (seven research articles and two reviews), look into the recent advancement and future trends related to the development of nanocomposites.

Carbon-based nanocomposites are an important class of functional nanomaterials for the remediation of environmental issues and the development of energy-related devices. Sun et al. engineered the dimensional interface of nanocomposite composed of BiVO_4_ and reduced graphene oxide (rGO) [1]. BiVO_4_ and rGO of different dimensions (OD, 1D and 2D) were synthesized and coupled to form a heterojunction semiconductor. It was observed that 2D–2D chemical coupling of BiVO_4_ and rGO exhibited the highest visible light activated photocatalytic activity toward the degradation of acetaminophen. The findings highlighted the importance of dimensional factors in the design of heterojunction photocatalysts. Lee et al. prepared mesoporous-rich activated polymer-based hard carbon to be used as electrodes in electrical double-layer capacitors [2]. The optimization of textural properties such as pore size and pore distributions was performed by altering the steam activation conditions to achieve the optimal electrochemical capacity. Cho demonstrated a facile method to synthesize low crystalline MoO_3_/carbon composite microspheres on a large scale [3]. Using a one-step spray pyrolysis technique, the MoO_3_ nanocrystals were evenly distributed within the amorphous carbon matrix. The resultant MoO_3_/carbon composite microspheres achieved higher Li-ion storage compared to MoO_3_ owing to their greater high structural stability and electrical conductivity.

The removal of heavy metals from wastewater using through adsorption and electrochemistry approaches has been investigated. Tan et al. reported an enhanced sludge treatment to remove Cu (II) ions followed by the application of the copper-doped porous materials as supercapacitor electrode materials [4]. The important aspects such as Cu (II) ion removal efficiency, specific capacity and cycling stability were evaluated. Silica-coated magnetic nanoparticles that were chemically functionalized with amino-rich polyethyleneimine (bPEI) have been developed by Plohl et al. and used as nanoadsorbents for Cu (II) ion removal [5]. The roles of bPEI in enhancing the performance of nanoadsorbents were discussed. The reusability and simple recycling of the nanoadsorbents were highlighted as the main features for practical applications for the treatment of heavy metal-contaminated sludge, sing polystyrene colloidal particles and GO as sacrificial template and reinforcing filler, respectively. Pan et al. prepared an aerogel consisting of porous calcium alginate and GO [6]. The nanocomposite exhibited high adsorption capacity towards Pb (II), Cu (II) and Cd (II) ions through ion exchange and chemical coordination mechanisms. Linh et al. incorporated titania nanoparticles (TiO_2_) into the 3D crossed region of stacked plasmonic Ag nanowires to enhance the photocatalytic activities [7]. In this nanocomposite, Ag nanowire contributed strong localized surface plasmon resonance excitation while the Ag nanowires–TiO_2_ interface rendered efficient hot electron transfer. The synergistic effects endowed the hybrid material with high photocatalytic performance across the ultraviolet and visible light spectral regions.

The two review articles compiled in this Special Issue were contributed by Subramaniam et al. [8] and Tu et al. [9], respectively. The former provided insights into the roles of nanomaterials in established and emerging technologies for hazardous heavy metal removal. The interesting features of nanomaterials and their hybrids as well as the synthesis techniques and removal principles are discussed in this review. Different heavy metal removal mechanisms, covering adsorption, filtration and oxidation using novel nanocomposites are presented. The second mini review looks into various in situ synthesis approaches for Si@C materials used for lithium ion batteries. With the increasing acceptance of Si@C materials for the development of lithium ion batteries due to their excellent lithium ion intercalation capacity and cyclic stability, this review presents the current progresses in the preparation and future research directions of Si@C materials.

The diverse contributions from these nine articles have witnessed the roles and importance of nanocomposites for environmental and energy applications. The versatilities of nanocomposites in addressing pollution and energy shortage issues have been evidenced. These contributions have addressed some of the important fundamental questions and shed light on the better understanding of the natures of the newly developed nanocomposite materials. It is hoped that this Special Issue could provide some useful guidelines for the future development of nanocomposites for environmental and energy applications.

## Data Availability

Not applicable.
